# Underwater Optical Imaging for Automotive Wading

**DOI:** 10.3390/s18124476

**Published:** 2018-12-18

**Authors:** Aleksandr Bystrov, Edward Hoare, Marina Gashinova, Mikhail Cherniakov, Thuy-Yung Tran

**Affiliations:** 1 Department of Electronic, Electrical and Systems Engineering, University of Birmingham, Birmingham B15 2TT, UK; e.g.hoare@bham.ac.uk (E.H.); m.s.gashinova@bham.ac.uk (M.G.); m.cherniakov@bham.ac.uk (M.C.); 2 Jaguar Land Rover Automotive PLC, Coventry CV3 4LF, UK; ttran3@jaguarlandrover.com

**Keywords:** advanced driver assistance systems, image analysis, object detection, optical propagation, optical sensors

## Abstract

An underwater imaging system was investigated for automotive use in highly scattered underwater environments. The purpose of the system is the driver’s information about hidden obstacles, such as stones, driftwood, open sewer hatches. A comparison of various underwater vision methods was presented by the way they are implemented, the range reached, and the cost of implementation. It has been experimentally shown that a conventional active system can provide a maximum visibility range of up to three light attenuation lengths. In most practical cases of turbid waters during floods, this corresponds to distances of about 1 meter. From the presented analysis it follows that advanced extended range imaging methods allow increasing of the visibility range up to 2 meters.

## 1. Introduction

**Figure 1:**
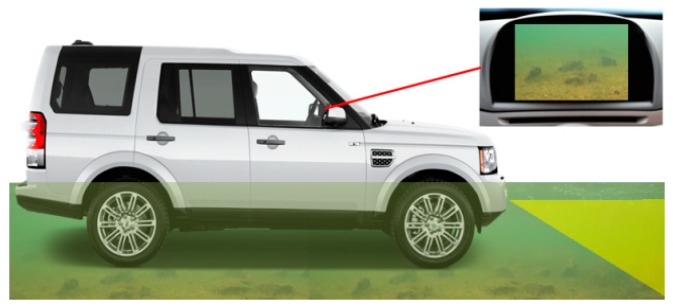
Automotive underwater imaging system.

In recent years the problem of flooding is set to increase in the UK with the onset of global warming. Large volumes of water can cause flash-floods, or floods in urban areas where the sewers and drains can’t cope. Therefore, the problem of driving safety in the case when the road is covered with a layer of water becomes more urgent. The ability of a car to maintain its stability and functionality when a vehicle is wading through deep water is crucial. Hidden objects underwater, including missing manhole covers, present one of the main dangers driving in a flooded urban environment especially when driving at night. In order to detect such objects, it is necessary to provide the driver with equipment that allows sensing through, and under, water (Figure 1). The optimum solution is to create an automatic system that recognizes hidden dangers. However, before developing such a system, we should understand what technologies can be used for underwater imaging and what range and accuracy they can provide. 

To the best of our knowledge, this paper represents the first such research in the field of automotive systems. However, we rely on the methods and results obtained in the development of sensing systems for submarines and other underwater vehicles. Studies in this area have been conducted for more than 50 years and there is a large body of work on this subject.

Modern vehicles sense their surroundings with different remote sensing techniques which include radar, sonar, and optical systems [1]. Radar can be utilized through fresh water, but it does not operate at high water conductivity, where salt or suspended solids are present. Sonar is widely used for underwater vision in water of any salinity; however it requires stable water to detect objects. The sonar signal is absorbed by muddy water and reflected and refracted by water turbulence. 

Light absorption and scattering in water are significant for optical wavelengths. Therefore optical imaging systems should be specifically designed to be used in underwater scenarios. The recent advances in hardware, software and algorithmic methods have led to significant improvement in image quality [2]. These advances primarily include the development of high-quality digital cameras in combination with effective image processing algorithms; introduction of compact, productive and inexpensive digital signal processors that provide ability to process streaming video using digital compression; the use of high repetition rate lasers and advanced time-gated detectors for two and three dimensional imaging systems. The modern imaging sensors that can provide dense information at high speed rates are commonly used for underwater applications. In this paper we will focus on the study of optical imaging methods, including LIDARs, in terms of their automotive use.

## 2. Water Optical Parameters

First of all, it is necessary to determine which parameters of water transparency we will use and establish a criterion for evaluating visibility in turbid waters. For this we consider the general laws governing the propagation of light in water and the methods for measuring the optical parameters of water.

Light power underwater can be described by Beer-Lambert law [2]:

(1) $$I(r)=I(r_{0})e^{-c(r-r_{0})},$$

where *I* is the intensity of light, *c* = *a* + *b* is the light attenuation coefficient (*a* is absorption, *b* is scatter), *r*_0_ is the transmitter position, and *r* is the receiver position. Both attenuation and absorption will depend on the type of particles and dissolved in the water substances.

The attenuation length *c*^−1^ is the distance where the intensity of the beam has dropped to 1/*e* of its initial value or about 63%. Clear waters absorb warm colors like reds and oranges (long wavelength light) and scatter the cooler colors (short wavelength light), so blue light penetrates the farthest. For turbid waters the penetration peak tends to shift towards the green-yellow waveband [3].

There are three common methods of characterizing water optical parameters [4]: total suspended solids (TSS), turbidity and clarity. TSS is a measurement of mass; they are reported in milligrams of solids per liter of water (mg/L). Turbidity is an optical determination of water clarity. To some extent, the turbidity for a given density of particles (TSS) is dependent upon properties of the particles such as their shape, color, and reflectivity. Turbidity can be measured with a turbidity meter and reported in units called a Nephelometric Turbidity Unit (NTU).

In our research we will also use measurements of water clarity based on the depth that a black and white Secchi disc can be lowered into a body of water. At the point visibility is lost, the Secchi depth *Z_SD_* is recorded. A horizontal measurement offers the ability to take a Secchi reading that is greater than the depth of the water. The horizontal visibility range can be calculated as [5]:

(2) $$Z_{SD}=\Gamma /c,$$

where Γ is the coupling constant. Although Γ can vary depending on the measurement conditions, it is generally accepted that for a black and white standard Secchi disk its average expectation is 1.7 [6]. Thus, knowing *Z_SD_*, we can characterize the visibility in terms of the number of attenuation lengths, which will allow us to compare the results obtained with the results of other authors.

## 3. Methods to Reduce Image Distortion

Obtaining high resolution underwater images remains a challenge due to image distortion. The most common forms of distortion in an underwater image are caused by water’s absorption of light and thus the subsequent decrease in intensity as the water depth increases. Another common form of distortion in an underwater environment is caused by scattering of a light source, be it a natural light source above the surface or an artificial light source situated within the body of water.

All underwater optical imaging systems can be divided into two classes: passive systems which utilize external light (sunlight) and active systems which utilize user generated source of light. Active systems can be conventional, when light source placed close to the camera, and extended range imaging systems. The purpose of extended range imaging systems is usually to mitigate the typical contrast limited in the conventional system due to volume scattering.

To reduce the common volume (FOV—field of view) several techniques or their combination can be applied [7,8,9,10,11,12]; they are presented in Table 1. The cost of the systems was estimated both from publications in open sources and through surveys of manufacturers of relevant equipment, including Hamamatsu Photonics (Hamamatsu, Japan), PHOTEK (St Leonards-on-Sea, UK), LaVision (Göttingen, Germany), Stanford Computer Optics (Berkeley, CA USA), and others.

The recent advances in underwater imaging have been discussed in [8]. In a range-gated system a source emits a pulse of light with the duration of several nanoseconds or less and the camera shutter waits for the time light takes to propagate from the emitter to the target, scatter in the target and back again to camera. Only the light scattered by the target is received and considered for imaging. These systems should have a very precise light gating in the camera receptor [10]. 

Laser line scan (LLS) underwater imaging is a serial imaging technique which involves the optical scanning of a narrow instantaneous field of view receiver in a synchronous fashion with a highly collimated laser source over a wide swath of a surface. LLS imaging is more difficult to implement than other methods, it requires important post-processing software and can be more expensive than a standard solution. 

A pulsed laser and gated detection scheme [11] has been effective on an underwater vehicle in rejection of the backscattered component. A system, described in [12], was based on the time-of-flight approach and the time-correlated single-photon counting technique. The depth profiles of targets were acquired at distances of up to 8 attenuation lengths.

## 4. Underwater Imaging for Automotive Use

As shown in the previous section, the most advanced systems provide approximately four times the range compared to the conventional image acquisition. However, this improvement is achieved due to a substantial (tens of times) rise in the cost of the system. In addition, ensuring the reliability and water tightness of the equipment in accordance with automotive standards will lead to its cost increase. Therefore, in the first stage of our study, the results of which are presented in this paper, we experimentally tested the automotive underwater vision system built on the basis of a conventional system, which can be implemented quickly and without significant costs. 

This system must warn of hidden objects underwater in advance with sufficient time reserve. As a starting point let’s assume that the speed of the car when driving on the water-covered road is limited to 5 km/h. To ensure a warning time interval of 2 seconds, the system must have a range of about 2.5 meters. Passive imaging systems are of little use in adverse weather conditions or in darkness. Therefore the developed system should be based on active approach, preferable use extended range imaging architecture. 

Modern automotive forward looking video cameras are usually placed at the highest possible height, which ranges from 1.5 m to 2 m. As will be shown later, placing the camera above the water level does not provide reliable recognition of underwater objects. Since the maximum wading depth for off-roaders usually does not exceed 0.5 m, the imaging system should be below that level. This roughly corresponds to the height of the fog lights (approximately 25–40 cm above the ground).

The light source and the camera are mounted on a vehicle; therefore the possibility to increase separation between them is limited by the car width (1.5–2 m). Let’s assume that we want to see a 2 m wide road band at a distance of 2.5 m. If the spotlight and the camera are close to each other, the angular width of the spotlight and the camera field of view should be approximately 44°. If they are separated by 2 m, this angle can be set to 38°, which will slightly reduce the common volume. A significant reduction in the FOV can be achieved by using narrow scanning beam of light synchronized with the camera.

Performance of the optical imaging system will depend on the brightness of generated light and camera sensitivity. The most efficient are illuminating systems based on xenon, LED and laser sources of generated light [9]. In our study, we used LED light sources which have high brightness with moderate power consumption, and many of them are waterproof.

According to some studies [9], objects in scene preserve polarization whilst backscatter do not, therefore polarization can be used to reduce the negative effects of backscatter. However, in the majority of such works, the case where the sun light is sufficient and no artificial illumination is required is investigated. Nevertheless, we investigated the influence of polarization on the visibility range of an active system.

A fairly simple way to increase system performance is to select the optimal wavelength of the emitted light. As already mentioned, green-yellow waveband is the optimal for turbid waters. In our research, we investigated light sources operating in the optical range from infrared to ultraviolet wavelengths.

## 5. Results of Laboratory Experiments

### 5.1. Camera and Light Source are above the Water

**Figure 2:**
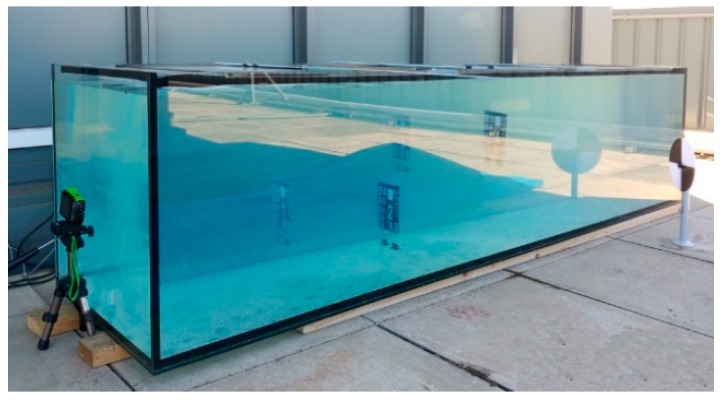
Water tank.

**Figure 3:**
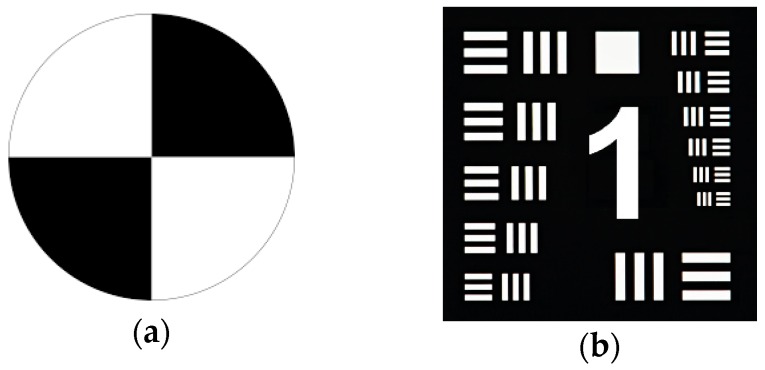
(**a**) Secchi disk; (**b**) Resolution test chart.

The controlled experiments have been conducted in a water pool and a large water tank (approximately 3 m long) with transparent glass walls (Figure 2). The water transparency was adjusted by adding colloidal liquid (Maalox). Two types of targets have been used in the experiment (Figure 3): standard black and white Secchi disk and modified 1951 USAF resolution test chart, which is a resolution test pattern conforming to MIL-STD-150A standard (Figure 2b). It is still widely accepted to test the resolution of optical imaging systems. Each test chart element consists of three horizontal and three vertical bars. The camera resolves a chart element, if the horizontal and the vertical bars can still be recognized as three distinct bars und don’t blur into one another. The size of the chart was 14 × 14 cm and the width of the bars decreased from the widest 3.7 mm to the narrowest 1 mm.

The first part of the experiment was the investigation of the ability to detect objects in the water when the camera and light source are located above the surface. This option has several advantages: it is not necessary to provide equipment waterproofness; the underwater beam path is shorter, especially if the system is installed high above the water surface, and hence the light absorption is lower.

**Figure 4:**
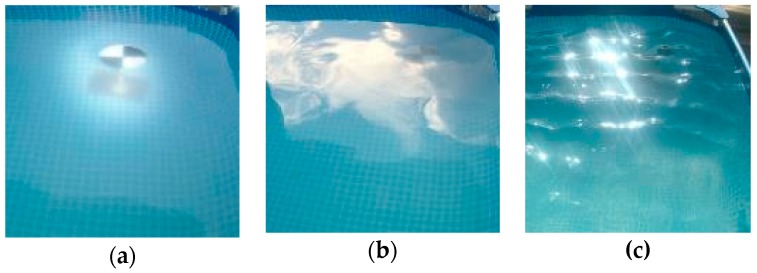
Camera and lamp are above the water: (**a**) dusk, calm weather; (**b**) daylight, calm weather; (**c**) daylight, windy weather.

In an image taken of an underwater target from above the surface of a body of water, the most common forms of distortion are caused by reflections of light off the surface of the body of water, which can obscure a target object or appear as a second object in a target detection, refraction resulting in a geometric deformation of the target and thus resulting in the loss of shape and causing the possibility of not being able identify or misidentifying a target object and also change in apparent position due to the change in refractive index of air and water. Indeed, the experiment showed that in bright sunlight underwater objects were difficult to see due to the mirror effect and during windy weather the objects were indistinguishable in rough water (Figure 4).

### 5.2. Test of Passive and Active Systems

**Figure 5:**
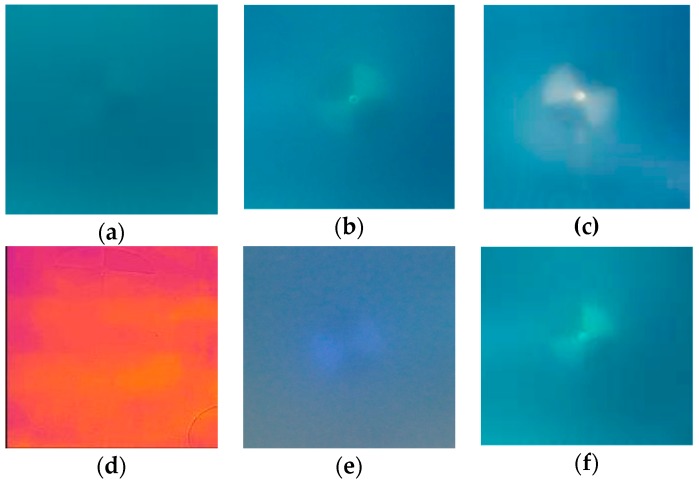
Secchi disk at 1.0 m distance: (**a**) passive imaging; (**b**) active imaging (1200 Lm); (**c**) active imaging (5000 Lm); (**d**) infrared imaging; (**e**) ultraviolet imaging; (**f**) polarized light.

Subsequent experiments were carried out in turbid water, the transparency of which was controlled by the addition of Maalox. For a passive system, the reference transparency was achieved by adding an amount of Maalox so that the horizontal Secchi distance *Z_SD_*, measured in the daytime, was 1 m (Figure 5a). Considering a real situation, we must take into account that the range of the reliable detection of low-contrast objects can be less than the Secchi distance.

The calculated light attenuation length *c*^−1^ based on this Secchi distance (2) was 0.60 m. This result was achieved by adding of approximately 80 mL of Maalox per cubic meter of water, which corresponds to TSS (aluminum and magnesium hydroxides) volume of 66 mg/L. The measured turbidity was equal to 4 NTU.

The Secchi distance relates only to a passive system. However, for active system we can expect that the object (Secchi disk) detection range will increase in accordance with Table 1 data. The photographs in Figure 5b,c confirm this assumption. Indeed, in an experiment involving Mares eos12rz LED flashlight with a brightness of 1200 lumens, the Secchi disk was distinguishable up to a distance of 1.30 m, which corresponds to 2.2 attenuation lengths. An ultra-bright Acebeam K60 LED flashlight (5000 lumens) allowed increasing the detection range of the object to 1.6 meters (2.7 attenuation lengths). 

The brightness of automobile headlights and fog lamps usually ranged from 1000 to 3000 lumens, sometimes reaching 5000 lumens. Therefore the LEDs flashlights used in the experiment can be considered as typical examples of such headlights.

Because the inanimate objects in the water normally have ambient water temperature, an infrared camera cannot distinguish them. This can be seen from Figure 5d, where the Secchi disk was lit by an infrared torch (5 W, 850 nm) and the photo was taken by FLIR ONE thermal imaging camera. 

As can be seen by comparing photos in Figure 5b,e, white light provides greater visibility than ultraviolet. At 1.0 meters the disk was visible in a white light (1200 lumens), but was on the verge of visibility in ultraviolet light. This is partly due to the fact that the white flashlight was brighter than the ultraviolet torch (10 W, 365 nm). As it will be shown in the following sections, blue colors (and, apparently, ultraviolet light) are not optimal in turbid waters.

As noted in the Section 4, the different behavior of objects and backscatter in front of polarized light can be used to improve contrast of underwater imaging. However, results of our experiments using various configurations of polarizing filters do not allow clearly asserting its advantage for underwater vision. We saw only a slight increase in the contrast of the image in some experiments (Figure 5f).

### 5.3. Experiment with Color Filters

Further measurements were performed using modified 1951 USAF resolution test chart. The advantage of using such a test before the Secchi disc is the ability to more accurately determine the range of distinguishing details of underwater objects. 

**Figure 6:**
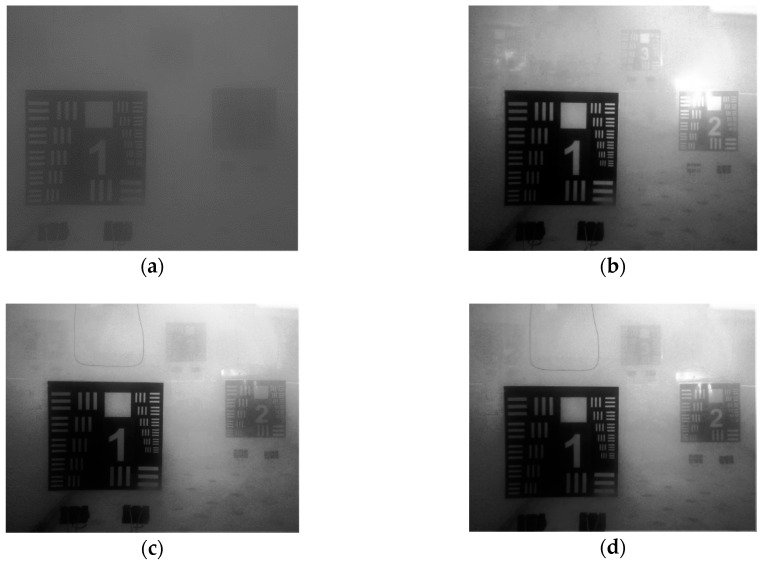

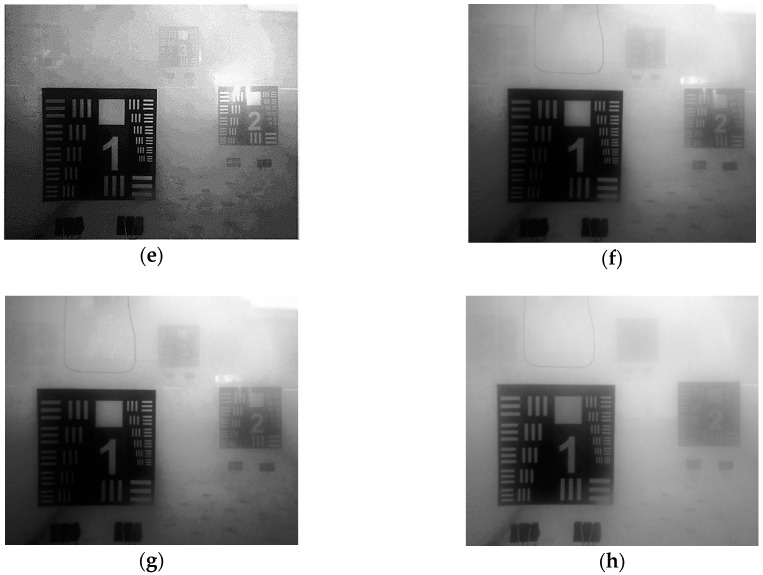
Resolution test charts: (**a**) passive imaging, (**b**–**g**) active imaging with 5000 Lm lamp: (**b**) white light (no filter), (**c**) red, (**d**) orange, (**e**) yellow, (**f**) green and (**g**) blue color filters; (**h**) ultraviolet lamp.

The color filters were attached to a flashlight: red (680 nm), orange (610 nm), yellow (580 nm), green (530 nm), and blue (470 nm). Figure 6 shows photographs taken using these filters. For a more accurate comparison, we converted these images into monochromatic shades of grey. The resolution test charts were placed at 0.5 m, 1.0 m, 1.5 m, and 2.0 m distances. Measurements were made on a bright, sunny day, but the water tank was in a shadow. As seen in Figure 6a, with passive measurement, the elements of the target 2 (1.0 m) are indistinguishable. When using a powerful torch (Figure 6b), the elements of target 3 (1.5 m) become readable. Target 4 at 2.0 m distance was almost invisible in all cases.

The highest penetration in the considered case is shifted to the green-yellow wavelengths, which is in accordance with the results of other researchers [12]. From comparison of target 3 photos it evidently follows that the best visibility is provided by white (Figure 6b) or yellow lights (Figure 6e), the orange light is slightly inferior to them (Figure 6d). Red, green and blue lights provide a lower visibility range of objects. 

It is necessary to take into account that the color filters weaken the light intensity by approximately 20% in comparison with the white beam. Therefore the white beam without filters is the brightest (but not the optimal). All this is additionally superimposed on the spectral response of a specific camera sensor, making it difficult to obtain accurate estimates. Nevertheless, in the experiments carried out using an ultra-bright torch in combination with yellow filter, the Secchi disk detection range was 1.8 meters (3 attenuation lengths). As noted in the paper earlier, white light provided a Secchi distance of 1.6 meters.

Similar to the previous experiment, ultraviolet light showed no advantages over other types of illumination (Figure 6h)

### 5.4. Image Post Processing

**Figure 7:**
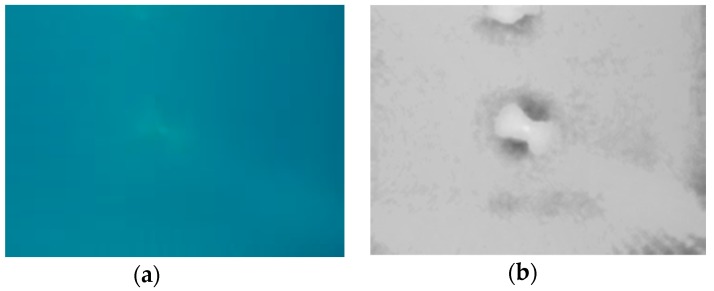
(**a**) Secchi disk image: (**a**) before processing; (**b**) after processing.

Beyond the scope of this study there was an important problem of improving object recognition by using image post processing, which includes de-noising and data processing techniques. Such methods as image enhancement and colour correction are well known [13]. They are capable of extending the range of underwater imaging, improving image contrast and resolution. As can be seen from the example presented in Figure 7, the use of these methods allows us to detect a Secchi disk that almost merged with the background on the original image. 

In [14] the detailed description and comparison of more advanced post processing techniques is provided. As shown in [14], the use of such methods as spatial, Fourier, wavelet processing, and sparse representation can significantly improve the identification of underwater objects. The advantage of this approach is the ability to extend the visibility range without significant increase in the cost of equipment.

## 6. Test under Close to Real Conditions 

Water flows can pick up and carry along mud and sediments that would reduce the transparency of the water [4]. Therefore usually the flowing water is more turbid than standing. We conducted relevant experiments in the tank by creating artificial turbulence of water. The experiment was conducted in the evening, to avoid the influence of sunlight on the results. However, we did not notice any significant change in the visibility of objects. Apparently this is due to the fact that in contrast to real water flows, in our case the amount of dissolved particles remained unchanged. The results obtained refer to the case of a colloidal solution and cannot be extended to a stream of water with larger particles of suspended matter.

**Figure 8:**
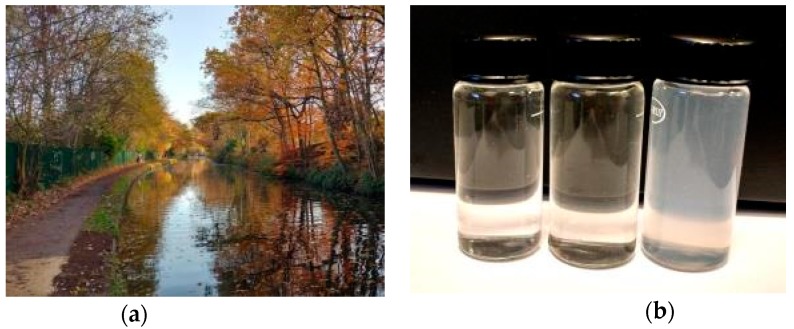
(**a**) Worcester and Birmingham canal; (**b**) water samples (left to right): 0 NTU (distilled water); 16 NTU; 100 NTU (calibration standard).

**Figure 9:**
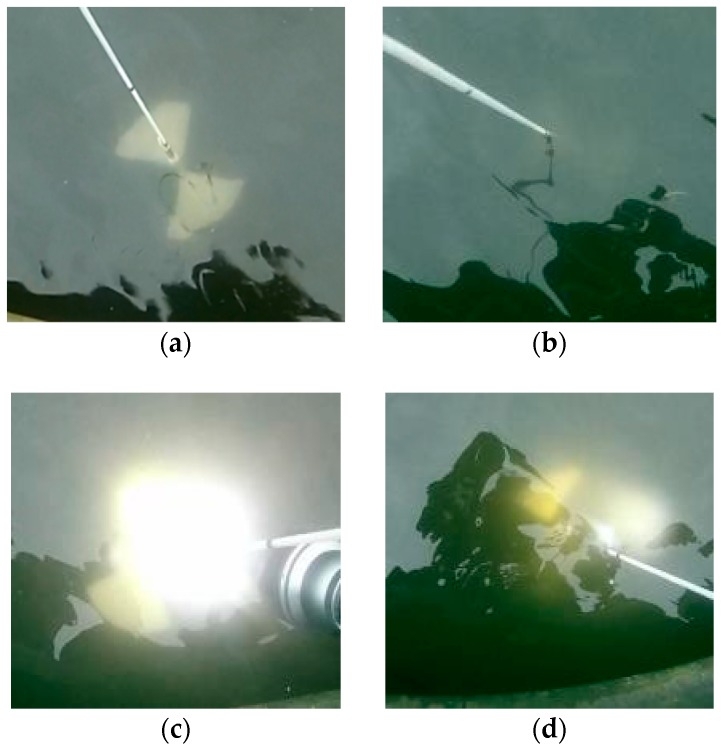
Secchi distance measurements at daylight: (**a**) passive 0.25 m, (**b**) passive 0.50 m, (**c**) active 0.25 m, (**d**) active 0.50 m.

The water in the Worcester and Birmingham canal (Figure 8) near the University of Birmingham has been tested. This water can be considered as an example of water during floods, and it seems completely opaque. However, measurements showed that water turbidity in the canal was equal to 16 NTU (Figure 8) and the vertical Secchi distance was 0.5 m (Figure 9). Light attenuation coefficient in this case is equal to 0.3 m. 

Based on the results, presented in Table 1, we can assume that visibility in canal using the conventional active imaging system will be about 0.8 m, and using extended range imaging system—up to 2 m. Indeed, when the Secchi disk was lit by 1200 lumen flashlight, it was visible to about 0.8 m (which is the depth of the canal near the bank). The use of a brighter flashlight can increase this distance, according to our estimates, to about one meter.

Most rivers and lakes are fairly clear with a turbidity reading below 10 NTU. These readings can easily jump into the hundreds due to runoff during a rainstorm, snowmelt or a dredging project [4]. However the typical value of the Secchi distance in rivers and lakes is several meters (2 m–3 m). This corresponds to a detection range of 3 m–4 m using a conventional active system and 8 m–12 m using an extended range imaging system. Tentative summary results are presented in the Table 2.

## 7. Discussion of the Results

We conducted a study of a conventional underwater vision system, based on a camera and a source of continuous light. The experiments showed that using a conventional system we can distinguish objects under water within the range which is determined by the following factors:

- Water turbidity;
- Type of natural light (for example, bright sun or darkness);
- Brightness and type of the light source;
- Color of the filter (the best results were achieved using yellow light).

Studies have also shown that infrared light source is ineffective for underwater vision. Ultraviolet light source is less effective than white of yellow. A noticeable effect of using polarized light in the experiments was not detected.

If we assume that the water during floods has the same turbidity (transparency) as the water in the canal (16 NTU), the conventional system can provide the distance of underwater vision up to one meter. The quality of recognition of individual objects will depend on their size, contrast, etc., and may be less than one meter. 

In this paper, only conventional imaging system was considered. However, as shown in Table 1, extended range imaging methods allow increasing of the visibility range up to 5–8 attenuation lengths. This corresponds to 3–4 Secchi depths. In a canal, where Secchi distance was 0.5 m, it gives 1.5–2.0 m visibility, i.e., up to two times more than a conventional system. Our future plans include experiments with increased separation between the light source and the receiver, and testing range-gated method in the laboratory conditions.

At present, range-gated and LLS systems look too expensive for automotive applications. However, with the progress of the corresponding technology and the reduction in the cost of the equipment, there will arise the opportunities for their installation on luxury all-road cars.

## Figures and Tables

**Table 1:** Imaging systems classification.

Type	Visibility ^1^	Cost, GBP
Passive imaging	1.7	<500
Active imaging: Conventional image acquisition	2.0–2.5	800
Active imaging: Extended range imaging		
Narrow FOV imaging	3	1000
Increased separation between the light source and the receiver	2.5–3	1000
Polarization discrimination: extracting the backscattering from images with different polarizations	2.5–3	1000
Time discrimination (range-gated methods): spatially broadened laser pulse as the illuminator and a non-scanning gated intensified camera as the detector	5	25,000–40,000
Spatial discrimination (laser line scan methods - LLS): optically scan a narrow instantaneous FOV receiver synchronously with a highly collimated laser source linearly over a large angle	5	20,000–50,000
LLS with increased source-receiver separation: increased source-receiver separation that reduces the scattering	6	20,000–50,000
Pulsed LLS imager with receiver gating: combination of LLS with time discrimination	>7	50,000–80,000
Time–correlated single–photon counting technique and point-to-point scanning	8	— ^2^

^1^ Attenuation lengths; ^2^ No cost information available.

**Table 2:** Visibility range.

	Canal	River	Lake	Sea
Secchi distance, m	0.5	2.0	3.0	10.0
Conventional active system, m	1.0	3.0	4.0	15.0
Extended range system, m	2.0	8.0	12.0	40.0
